# Simulation-Based Trajectory for Non-Planar Scaffold Printing on Irregular Patches Using Robotic Arm

**DOI:** 10.3390/bioengineering13030260

**Published:** 2026-02-24

**Authors:** Salvatore D’Alessandro, Gianluca Cidonio, Giancarlo Ruocco, Franco Marinozzi, Fabiano Bini

**Affiliations:** 1 Department of Mechanical and Aerospace Engineering, University of Rome “La Sapienza”, 00184 Rome, Italy; salvatore.dalessandro@uniroma1.it (S.D.); gianluca.cidonio@uniroma1.it (G.C.); franco.marinozzi@uniroma1.it (F.M.); 2 Center for Life Nano- & Neuro-Science—CLN2S, Italian Institute of Technology (IIT), 00161 Rome, Italygiancarlo.ruocco@iit.it (G.R.)

**Keywords:** bioprinting, non-planar scaffold, tissue regeneration, robotic arm

## Abstract

This study proposes a reproducible and accessible methodological framework for non-planar path generation to enable scaffold biofabrication on irregular anatomical surfaces replicating the native morphology of human tissue. By integrating a simulation-based trajectory optimization system with a robotic arm, lattice paths are generated using an intersection-based method with parallel planes. This method is processed by intersecting the anatomical object with orthogonal planes, allowing for the creation of paths that conform to complex geometries. The proposed approach relies on widely available and commonly used tools, such as MATLAB, avoiding the need for highly specialized software. Thus, a MATLAB-based kinematic model computes optimal end-effector trajectories, while a coaxial nozzle facilitates the simultaneous extrusion of an alginate-based biomaterial. The proposed method ensures smooth trajectory execution, achieving positional standard deviation within the reproducibility threshold of the robotic arm for an optimal path discretization density. Unlike conventional planar methods, the optimized approach achieves positional accuracy within the robotic arm’s reproducibility threshold while demonstrating superior geometric conformity on complex anatomical patches. The approach successfully fabricates scaffolds with controlled deposition on anatomical patches, demonstrating improved geometric conformity over traditional planar methods. This method provides a pathway for patient-specific scaffold fabrication, supporting advances in tissue engineering and regenerative medicine.

## 1. Introduction

The development of innovative methodologies for scaffold fabrication has become a central focus in the field of tissue engineering and regenerative medicine [1,2]; it involves developing biological models to mimic the physiologic behavior of tissue [3,4,5]. The core of the tissue engineering and regenerative medicine (TERM) field is the fabrication of scaffolds that perform as temporary frameworks for supporting proliferation [6,7] and differentiation [8,9,10] while simultaneously guiding tissue regeneration [11,12,13]. Specifically, scaffold geometry is essential for ensuring that the scaffold fits the anatomical shape of the mimicked tissue [14,15]. In fact, traditional 3D printing approaches rely on planar deposition, which is limited in reproducing the complex geometries of anatomical structures. Thus, many biological tissues, such as bones, cartilage, and certain organs, have highly irregular and non-planar surfaces that cannot be accurately replicated using planar strategies [16,17,18]. While layer-by-layer extrusion dominates scaffold fabrication due to its simplicity and compatibility with a wide range of bioinks, planar deposition inherently produces stairstep artifacts on curved surfaces and fails to conform to highly irregular anatomical geometries, resulting in poor cell seeding efficiency and suboptimal mechanical integration [19,20]. Existing non-planar strategies address this limitation through surface contouring, robotic freeform deposition, or five-axis CNC machining, but they typically require specialized software (e.g., Autodesk PowerMill, Siemens NX), custom G-code generators, or high-end industrial robots, limiting accessibility for academic labs [21,22]. In contrast, our MATLAB-based intersection method leverages standard STL slicing with parallel planes to generate lattice toolpaths on any anatomical patch, using widely available desktop robotic arms like the DOBOT MG400. This approach combines the simplicity of planar slicing with true surface conformity, without proprietary software dependencies. To address this challenge, new techniques for non-planar path generation are needed, enabling the fabrication of scaffolds that conform to complex anatomical geometries. Non-planar bioprinting strategies offer a transformative approach to scaffold fabrication, allowing for the creation of patient-specific scaffolds with improved surface conformity and geometric complexity [23,24]. These methods involve generating paths that follow the contours of irregular surfaces, thereby enhancing the precision of biomaterial deposition. Such approaches are particularly relevant in regenerative medicine, where the ability to fabricate anatomically accurate scaffolds plays a crucial role in achieving effective tissue repair and functional recovery [13,14,15].

This study introduces a methodology for the design of non-planar paths for scaffold fabrication on irregular anatomical surfaces. The approach is based on a simulation-driven process that integrates a kinematic model of a robotic arm with an optimized trajectory generation algorithm both reducing the computational cost and adapting the path resolution to robotic arm features. Moreover, the proposed method employs an intersection-based strategy to generate lattice paths on the surface of anatomical patches. This process ensures precise control of the end-effector (EE) and facilitates the deposition of biomaterials on irregular surfaces. The trajectory design process is validated using a robotic system equipped with a DOBOT MG400 robotic arm, which is guided to deposit an alginate-based biomaterial onto a support replicating an anatomical surface. While traditional planar methods excel in simplicity, speed, and established protocols for flat or gently curved surfaces, they fail to conform to highly irregular anatomical geometries, resulting in poor surface adaptation and air gaps. Our accessible intersection-based approach overcomes these limitations by generating lattice toolpaths that precisely follow complex 3D surface topography using widely available MATLAB tools, without requiring specialized software.

It is important to emphasize that the primary objective of this work is to present a methodological framework for the generation of non-planar lattice paths on irregular anatomical surfaces, with a focus on trajectory design through surface slicing and spatial discretization. The lattice structure is presented solely as a demonstrative application of the proposed path generation method, and its functional performance is not intended to be isolated from other system components in this study. The study is not intended to address aspects related to optimal robotic motion planning, such as the minimization of joint angle changes, collision avoidance, or the implementation of a comprehensive inverse kinematics analysis with associated motion constraints. These elements, while relevant for real-time execution and motion efficiency, fall outside the scope of the present study. Instead, the aim is to propose a reproducible and adaptable strategy for non-planar path generation, laying the foundation for future research on robotic motion optimization and its integration with surface-based deposition techniques.

## 2. Materials and Methods

### 2.1. Materials

Ru-Tris (2,2′-bipyridyl) dichloro-ruthenium(II)hexahydrate (cat no. 224758, Sigma-Aldrich, 3050 Spruce Street, Saint Louis, MO, USA), sodium persulfate (SPS) (cat no. 216232, Sigma-Aldrich, USA), gelatine—type A3 porcine skin (~300 g bloom) (cat no. G1890, Sigma-Aldrich, USA), and methacrylic anhydride (cat no. 276685, Sigma-Aldrich, USA) were used. Laponite^®^-XLG was provided by BYK, Abelstraße 45, 46483 Wesel, Germany. Alginate (medium viscosity, Sigma-Aldrich, USA) was also employed.

### 2.2. Synthesis of Gelatine Methacryloyl (GelMA)

Gelatine methacryloyl (GelMA) was synthesized following previously reported protocols [25,26,27]. Briefly, gelatine was dissolved at a 10% (*w*/*v*) concentration in PBS (pH 7.5/8) at 50 °C. Methacrylic anhydride (MA, 0.8 mL per g of gelatine) was added to the gelatine solution dropwise under vigorous stirring for 3 h. Following this step, the solution was dialyzed against deionized water (DW) using 1–2 kDa cut-off dialysis tubes for ~5 days at 50 °C. Finally, GelMA was lyophilized and kept at 4 °C for further use.

### 2.3. Preparation Inks Based on Alginate-GelMA and Nanocomposite Inks

The ink blend, referred to as n-AG, was prepared using alginate (A), GelMA (G), and nanoclay (n, Laponite^®^) at concentrations of 4% (*w*/*v*), 1.5% (*w*/*v*), and 0.5% (*w*/*v*), respectively. The preparation followed previously reported protocols [28,29]. Materials were sterilized under UV light for 30 min prior to ink creation. Laponite^®^ was gradually added to deionized water (DW) under continuous stirring. After 2 h, alginate and GelMA were dissolved in the colloidal suspension at 40 °C with stirring for an additional 2 h.

### 2.4. Printing Path Generator

**Figure 1:**
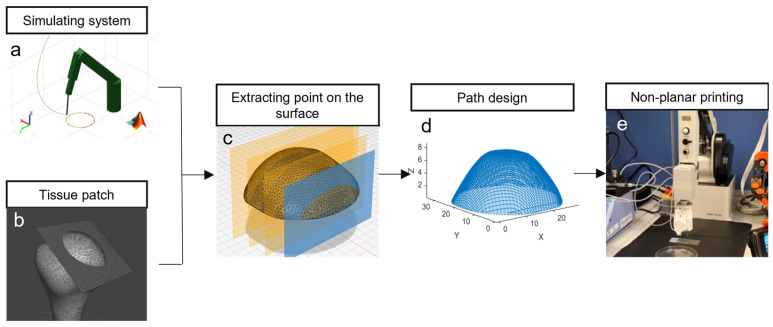
The figure outlines the flowchart of the proposed methodology for the creation of non-planar paths for irregular scaffold printing on irregular surfaces. (**a**) Development of a MATLAB simulation model that replicates the kinematics of the real robotic arm to optimize the trajectory creation for arm guidance. (**b**) Selection of an anatomical tissue patch for printing. (**c**) Extraction of points on the surface of the tissue patch by intersecting the object with parallel planes using custom MATLAB software. (**d**) Generation of the lattice path using custom MATLAB software on the points extracted in the previous step. (**e**) Guidance of the DOBOT MG400 robotic arm (provided by Shenzhen Dobot Corp Ltd., Shenzhen, China) along the extracted path with simultaneous extrusion of alginate-based biomaterial using a custom coaxial nozzle for non-planar deposition.

A MATLAB 2022b script was developed to generate the end-effector (EE) path of the robotic arm on an anatomical district, as shown in Figure 1. The method relies on extracting the intersection points of the object surface with parallel planes oriented in two orthogonal directions. By ordering the points on each plane along two perpendicular directions, a lattice path was generated on the irregular anatomical surface. This approach enables the intersection of STL files with parallel planes to obtain a lattice path on the surfaces for biomaterial deposition. The distance of the parallel planes is 1 mm and the distance among the path points is less than the robotic arm accuracy, allowing for precise control of the printing system. The selection of a 1 mm interplanar distance was adopted as a representative and demonstrative design parameter for the proposed non-planar path generation methodology.

The anatomical geometry was originally provided as a standard binary STL file (e.g., humeral head model, as shown in Figure 1b). This STL file was directly imported into MATLAB and the file was automatically parsed; the generated mesh is composed of vertices and triangular faces. This mesh serves as the input for the intersection-based path generation algorithm, where parallel planes spaced 1 mm apart intersect the triangles to extract surface contours for lattice toolpath creation in two orthogonal directions (Figure 1c).

The scaffold is printed with a grid-like pattern, where the filaments are deposited in alternating parallel layers to form a lattice structure. To generate this, the STL surface is intersected with parallel planes in two orthogonal directions. The intersection between each plane and the anatomical surface yields a set of linear paths, which are subsequently ordered and combined to form the complete deposition path. This method ensures a controlled filament arrangement that mimics the typical lattice infill used in tissue scaffolds, promoting mechanical stability and structural regularity.

The path generation proceeds intuitively from familiar STL slicing principles. Parallel planes spaced 1 mm apart slice through the anatomical patch’s triangular mesh in two orthogonal directions, creating linear intersection contours on the surface. Along each contour, points are sampled and ordered sequentially to form continuous path segments.

These segments form a lattice when X- and Y-direction families are interleaved, with scanning direction alternating between planes (left-to-right, then right-to-left) to minimize travel. Plane-to-plane jumps use the shortest 3D vector between endpoints, ensuring smooth execution. Complex curvatures are inherently handled as STL mesh density increases intersection points in these regions, providing natural path adaptation without special algorithms.

### 2.5. 3D Deposition of Biomaterial Ink

The printing process was conducted using the robotic arm (DOBOT MG400) with four degrees of freedom (DOF) and a repeatability of 0.05 mm. The device is equipped with a custom coaxial nozzle previously developed [28,29] with inner and outer diameters of 25 G and 18 G, respectively. The system is integrated with a syringe pump (Legato^®^ 101 Syringe) using an ink flow rate of 50 µL/min and a feed rate of 10 mm/s. Moreover, a 0.33 M CaCl2 solution along with ruthenium (Ru) at a concentration of 0.3 mM/mL and sodium persulfate (SPS) at 3 mM/mL were employed for crosslinking during the printing process.

### 2.6. Simulator Model

The kinematic simulation replicates DOBOT MG400 motion using standard Denavit–Hartenberg parameters, matching manufacturer link lengths L1–L4 and joint rotation axes. Forward kinematics maps joint angles to end-effector pose; inverse kinematics computes required joint configurations for each trajectory waypoint.

Joint limits follow device specifications but are not actively enforced, as paths were manually constrained to the verified central workspace. Collision detection and dynamic effects (backlash, compliance) were omitted to focus on spatial discretization optimization—the core methodological contribution. Trajectory quality is quantified solely as end-effector positional deviation from the analytical ideal path, with the benchmark circular trajectory providing a geometry-independent accuracy metric.

The model does not include collision detection or self-intersection prevention, and feasibility is ensured by restricting the generated paths to regions of the workspace known to be reachable by the robotic arm. Error propagation was evaluated exclusively in terms of positional deviation of the end-effector, assuming ideal actuation and neglecting dynamic effects such as backlash, compliance, or time-dependent disturbances. Under these assumptions, the simulation was used to verify that the spatial discretization of the path results in trajectory deviations below the mechanical repeatability threshold of the robotic system.

## 3. Results

This study presents an innovative methodology for non-planar guidance in the printing of biomaterials. The approach enables the optimization of the printing trajectory according to the specific printing system based on robotic arm and the irregular anatomical object to be reproduced. Figure 1 schematically illustrates the overall workflow with the key phases for the development of a non-planar path for scaffold printing on irregular surfaces. Thus, a MATLAB-based simulation model (Figure 1a) was developed to replicate the kinematics of the DOBOT MG400 robotic arm. The model allows for the prediction and optimization of the trajectory to be executed by the robotic arm. By simulating the movements of the robotic arm, the system enables the pre-validation of the trajectory to ensure a smooth, accurate, and optimized printing process. Simultaneously, an anatomical district is selected (Figure 1b), isolating the targeted printing region, which serves as the basis for defining the non-planar printing surface. The patch is extracted from the 3D model of the humeral head district represented as an STL file. Then, the relevant surface points are extracted from the anatomical patch district along two orthogonal directions for creating a lattice path (Figure 1c). The points are identified and recorded, intersecting the STL file, with parallel planes generating a point cloud on the surface of the patch. This step forms the basis or the construction of the non-planar path, which identifies the surface topography to define the printing trajectory. Subsequently, the extracted points are ordered for creating the printing lattice trajectory (Figure 1d), improving the spatial distribution, with generated simulated data reducing the computational cost. Finally, the printing phase involves the guidance of the DOBOT MG400 robotic arm along the extracted and optimized path (Figure 1e), with simultaneous extrusion of n-AG using a custom coaxial nozzle. These key phases provide a comprehensive framework for the generation of non-planar paths that improve the printing process for irregular anatomical surfaces. The following sections present the result of each step in greater detail.

### 3.1. Simulating Custom Software

**Figure 2:**
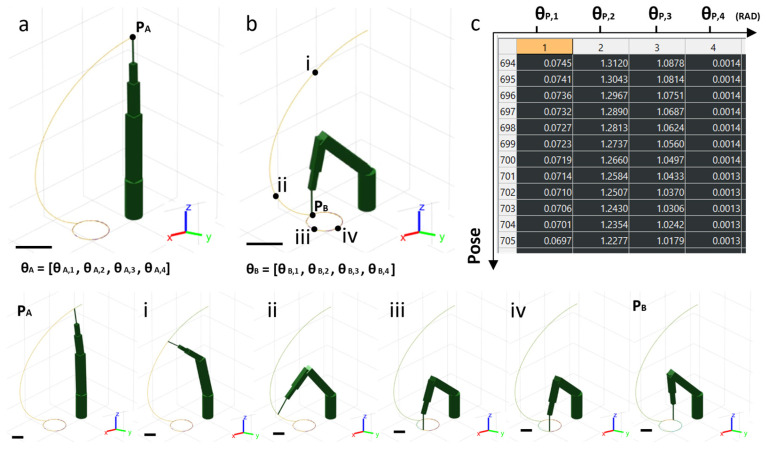
The figure illustrates the simulation model of the robotic arm, replicating the DOBOT MG400 during the path from the starting point P_A_, along a 20 mm circumference, to the endpoint P_B_. (**a**) The initial configuration of the arm is shown, with the arm in an upright position where the four joints have specific angles θ_A_ = [θ_A1_, θ_A2_, θ_A3_, θ_A4_]. (**b**) The final configuration of the robotic arm at position P_B_ is displayed, with specific joint angles θ_B_ = [θ_B1_, θ_B2_, θ_B3_, θ_B4_], after completing the movement from P_A_ to P_B_ through the stages i–iv. (**c**) This panel illustrates how the simulation system calculates the joint angles for each pose assumed by the robotic arm during the execution of the path. Scale bar: 10 mm.

A simulation system was developed using MATLAB software, incorporating the physical principles of direct and inverse kinematics. This approach enabled the calculation of the end-effector (EE) position and the corresponding joint angles at every point along the trajectory. The model was arranged for the geometry of the robotic arm, including link lengths and the rotation direction of the joints. The simulated robotic arm, shown in its upright position P_A_ in Figure 2a, displays the robotic arm links in green. Each joint is characterized by a specific joint angle θ. The first simulation involved movement from the rest position P_A_ to position P_B_, following a circular trajectory with a diameter D of 20 mm (Figure 2b). A circular trajectory was intentionally selected as a benchmark case because it provides an analytical reference geometry, enabling a quantitative evaluation of spatial discretization accuracy, while such reference metrics are not directly definable for complex non-planar anatomical surfaces. Every point along this trajectory corresponds to a distinct arm configuration, defined by a unique set of joint angles θ (Figure 2c). To improve the spatial discretization required for the trajectory, the circular path was used as a reference for the analysis.

**Figure 3:**
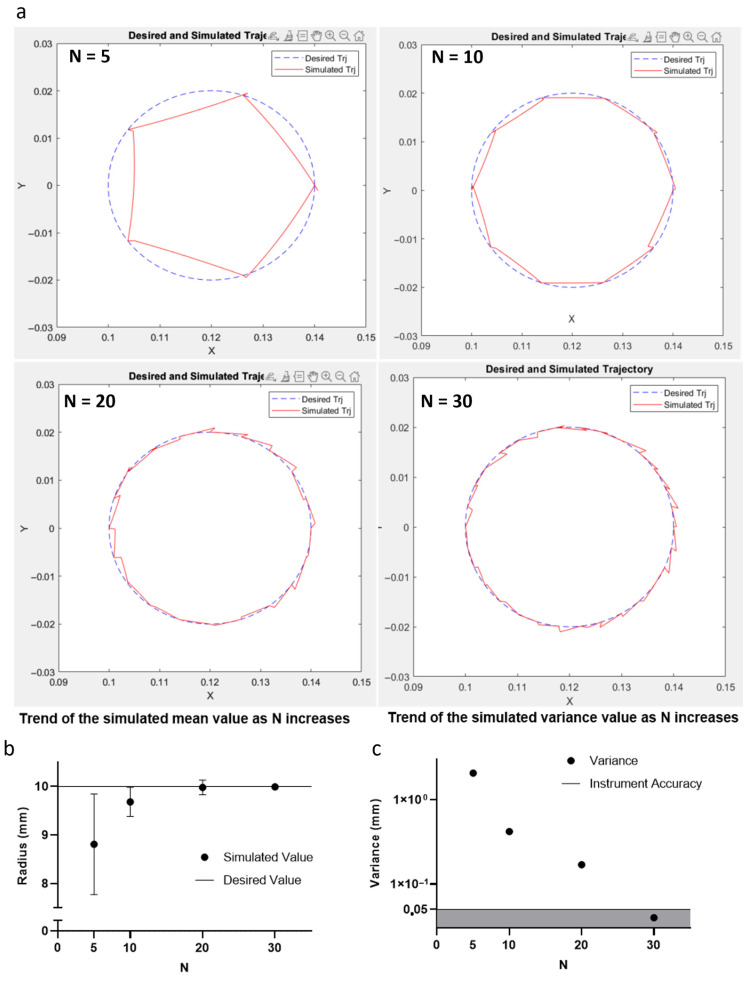
The figure illustrates the ideal trajectories (in blue) and simulated trajectories (in red) around a circumference with a diameter of 20 mm. (**a**) Simulated trajectories are shown for varying spatial discretization densities (N) with values of 5, 10, 20, and 30. (**b**) The graph displays the ideal radius value of 10 mm as a continuous line, while the points of the simulated trajectory exhibit different values depending on N. The points converge toward the ideal value as N increases. (**c**) The graph shows the trend of the measured variance of the points along the ideal trajectory, which decreases as N increases, and after N = 30, the variance is below the area subtended by the continuous line, representing the accuracy of the DOBOT MG400 device.

Different spatial discretization types (N = 5, 10, 20, and 30) were evaluated to guide the EE along the trajectory, as illustrated in Figure 3a. For each simulated trajectory, the mean radius value and the standard deviation were calculated.

The corresponding results are shown in Figure 3b,c, while the specific values are provided in Table 1. Moreover, Figure 3c also highlights a shaded gray area representing the reproducibility limit of the DOBOT MG400 robotic arm, which was used to improve the printing process. Finally, the spatial discretization density S can be calculated as follows:

S = N/πD(1)

The spatial discretization density (S) was improved through a kinematic simulation model to ensure that the positional standard deviation of the generated trajectory remains below the mechanical repeatability threshold of the DOBOT MG400 robotic arm, which is ±0.05 mm. By systematically increasing the number of trajectory points and evaluating the resulting deviation from the ideal path, we identified that a spatial sampling interval of 2.09 mm allows the system to achieve a positional absolute standard deviation of approximately 0.02 mm. This value confirms that the simulated trajectory is reproduced by the robotic arm with a level of accuracy finer than the mechanical resolution of the device, thus enabling highly controlled, non-planar motion of the end-effector during biomaterial deposition.

**Figure 4:**
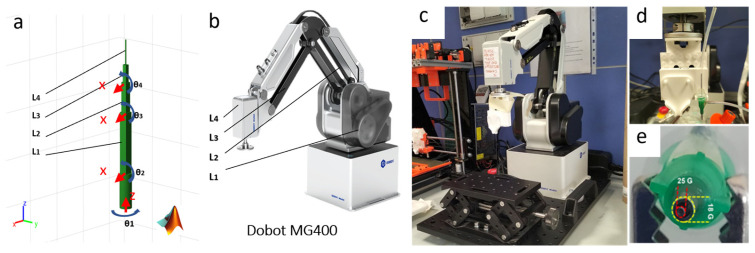
The figure illustrates the virtual and real printing system with the end-effector equipped with a print head. (**a**) The simulation model of the robotic arm is shown, highlighting the L1–L4 links, which have the same dimensions as those of the DOBOT MG400, and the joint rotation axes Θ1–4, identical to those of the real system. (**b**) Image of the device. (**c**) Photograph of the DOBOT MG400 robotic arm. (**d**) End-effector equipped with a print head, consisting of a LEGO-based support to which customized tools can be attached. (**e**) Image of the extrusion outlet, showing the coaxial nozzle with an inner and outer nozzle of 25 G and 18 G, respectively.

Simultaneously, the simulation model was further adapted to match the physical configuration of the DOBOT MG400 robotic arm. The link lengths and joint rotation axes were defined to align with the specifications of the physical device, as shown in Figure 4a,b. This adaptation ensured that the simulated kinematics were consistent with the real-world behavior of the robotic arm. The physical printing system was constructed using the DOBOT MG400 robotic arm mounted on a Thorlabs base to provide a stable and precise support structure (Figure 4c). The EE was equipped with a LEGO-based support (Figure 4d) designed to accommodate a custom coaxial nozzle (Figure 4e). The coaxial nozzle features two concentric nozzles with diameters of 25 G and 18 G. These nozzles enable the simultaneous extrusion of n-AG biomaterial and a 0.33 M calcium chloride aqueous solution.

### 3.2. Printing Path Design and Deposition

**Figure 5:**
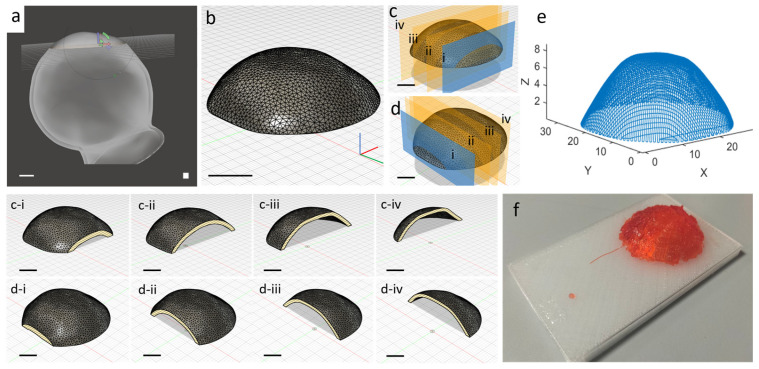
The figure illustrates the methodological process from the tomographic object of the anatomical district to printing, including the creation of the printing path. (**a**) STL file of a humeral head with a selected patch being extracted. (**b**) Section of the tissue district showing the points that compose the external surface of the object. (**c**,**d**) Methodology used to extract the lattice path on the external surface of the anatomical district. The path was obtained by intersecting the object with parallel planes in two orthogonal directions, as shown in c_i–iv_ and d_i–iv_. (**e**) Lattice path extracted using MATLAB software. (**f**) Image showing the successful deposition of n-AG on a printing support with the same geometry as the selected anatomical district. Scale bars: 5 mm.

The design of the printing path begins with the selection of an anatomical tissue patch extracted from an STL file of a humeral head. The patch was isolated by sectioning a portion of the tissue using a cutting plane, resulting in a circular region with a diameter of approximately 20 mm (Figure 5a). The isolated tissue patch is extracted from a standard binary STL file of a humeral head (0.05 mm resolution; Figure 5a) and imported directly into MATLAB, converting the file into a computational triangular mesh structure (Figure 5b). While STL files provide static triangle geometry for visualization/3D printing, the MATLAB mesh enables dynamic plane–triangle intersection algorithms essential for path generation (Section 2.4). Parallel planes (1 mm spacing) then slice through these mesh triangles to extract surface contours for lattice toolpath creation (Figure 5c,d).

Figure 5f confirms that the simulation-optimized trajectory (S = 2.09 mm) executes successfully on physical hardware, with n-AG filaments visually conforming to the curved anatomical surface. This qualitative demonstration validates the non-planar path generation framework. Quantitative geometric fidelity analysis and scaffold performance metrics will be addressed in future studies. The value of S was set to ≤2.09 mm, corresponding to the optimal spatial discretization density (N = 30) previously determined using the kinematic simulation system (Table 1), ensuring that the absolute positional standard deviation remained below the DOBOT MG400 robotic arm’s 0.05 mm repeatability threshold.

These intersection planes were oriented along two orthogonal directions to generate linear paths, which were combined to create a lattice structure. The interplanar distance was set to 1 mm, thereby creating a reticulated structure with 1 mm pores. Along each intersection line between the plane and the surface of the patch, a spatial discretization density (S) was applied. The simulation-optimized spatial discretization S = 2.09 mm (N = 30, Table 1: σ = 0.02 mm < 0.05 mm repeatability) was directly applied to generate the anatomical lattice path (Figure 5e), ensuring that the physical printing trajectory (Figure 5f) inherits the validated positional accuracy. This strategy allows for a distribution of filaments over an irregular anatomical surface while maintaining geometric conformity. The interlayer pattern alternates direction between consecutive layers to achieve a consistent pore structure throughout the scaffold. By carefully tuning the interplanar spacing and the spatial discretization along each path, the methodology ensures that the trajectory is accurately followed by the robotic arm. This strategy ensures precise filament deposition over the irregular anatomical surface while visually maintaining geometric conformity to the target patch morphology (Figure 5f). The trajectory accuracy is guaranteed by the simulation-optimized spatial discretization (S ≤ 2.09 mm), which achieves positional deviations below the robotic arm’s repeatability threshold. Quantitative assessment of scaffold geometric fidelity relative to the target STL surface will be addressed in future validation studies.

Once the surface points were extracted, they were ordered to generate the printing path, as shown in Figure 5e. This path served as the trajectory for the robotic arm to guide the deposition of n-AG on a support replicating the geometry of the irregular anatomical patch. The integration of the optimized printing path with the robotic arm system enabled the precise deposition of biomaterials on complex anatomical surfaces, supporting the fabrication of scaffolds with non-planar geometries.

## 4. Discussion

This study proposes an accessible approach for non-planar path design for the deposition of biomaterials on irregular anatomical surfaces. By integrating a simulation-based trajectory optimization system with a physical robotic arm, significant advancements in the optimization of the printing path were achieved. The kinematic simulation results (Section 3.1, Figure 3, Table 1) demonstrate that increasing the spatial discretization density N from 5 to 30 points progressively reduces the positional standard deviation σ of the end-effector trajectory from the ideal reference path. Specifically, σ decreases to 0.02 mm at N = 30, remaining below the DOBOT MG400’s 0.05 mm repeatability threshold. This N = 30 configuration (corresponding to S ≈ 2.09 mm along a 20 mm reference path) represents the optimal trade-off: further increasing N provides negligible accuracy gains (σ already < mechanical limit) while proportionally increasing computational cost (path generation time scales linearly with N) and data volume for robotic arm execution. Thus, adopting S ≤ 2.09 mm ensures printing accuracy within hardware constraints while minimizing unnecessary computational overhead. The kinematic simulation results (Figure 3, Table 1) demonstrate that increasing the spatial discretization density N from 5 to 30 points progressively reduces the positional standard deviation σ of the end-effector trajectory from the ideal reference path. Specifically, σ decreases to 0.02 mm at N = 30, remaining below the DOBOT MG400’s 0.05 mm repeatability threshold. This N = 30 configuration (corresponding to S ≈ 2.09 mm along a 20 mm reference path) represents the optimal trade-off: further increasing N provides negligible accuracy gains while proportionally increasing computational cost and data volume for robotic arm execution. Thus, adopting S ≤ 2.09 mm ensures printing accuracy within hardware constraints while minimizing unnecessary computational overhead. Furthermore, determining an optimal value for S prevents the need for an excessively high spatial density, which would unnecessarily increase computational cost, while also avoiding a low spatial density, which would significantly reduce the quality of the printing path. This approach ensures the precise execution of non-planar paths with optimized computational cost. It is important to note that this study is primarily intended as a methodological contribution focused on non-planar path generation. Therefore, statistical analyses, repeatability testing, and quantitative comparisons between planar and non-planar deposition strategies were not addressed. These aspects, while essential for a comprehensive assessment of printing performance, fall outside the scope of the present work and will be explored in future experimental studies. This methodological study intentionally prioritizes trajectory optimization over exhaustive scaffold characterization. While simulation confirms positional accuracy within hardware limits, printed results demonstrate feasibility rather than optimized scaffold performance. Planned future work includes quantitative deviation mapping, printing repeatability assessment, and controlled planar vs. non-planar comparisons.

Additionally, the proposed methodology enables the creation of lattice-like paths on anatomical patches, resulting in the successful deposition of an n-AG biomaterial scaffold on a 3D surface.

The proposed approach for non-planar path design was validated through simulation and execution of trajectories on a humeral head anatomical patch. The MATLAB-based simulation model demonstrated that a higher spatial discretization density (S) results in lower positional standard deviation along the path. Notably, the absolute standard deviation observed for N = 30 was within the positional accuracy threshold of the DOBOT MG400 robotic arm, confirming that this density is sufficient to ensure precise guidance of the end-effector. This optimization process guaranteed smooth trajectory movement, facilitating accurate path execution.

The simulation of the robotic arm movement from position Pa to Pb, as shown in Figure 2, underscored the effectiveness of the trajectory design. Each pose of the robotic arm was characterized by specific joint angles, enabling the end-effector to follow a circular path with a diameter of 20 mm. The results demonstrated that the positional standard deviation along the trajectory decreased as the density of spatial discretization points increased, as shown in Figure 3b,c. These findings are further supported by Table 1, which presents the mean radius values and absolute standard deviation for each discretization density.

The jagged appearance observed in the simulated path under low spatial discretization conditions is a known limitation. However, the problem was automatically resolved by increasing the number of discretization points, which leads to a smoother and more accurate representation of the ideal path. As demonstrated in our analysis, a discretization density of N = 30 results in a simulated trajectory absolute standard deviation lower than the repeatability threshold (±0.05 mm) of the DOBOT MG400 robotic arm, thereby ensuring that such fluctuations do not affect the actual motion during robotic execution. This guarantees precise end-effector guidance without introducing errors in the printing process.

The findings of this study align with prior research on non-planar path generation but introduce an accessible intersection-based approach for path design. The proposed method employs parallel planes in two orthogonal directions to extract points from the anatomical patch, enabling the generation of lattice paths that conform to the irregular surface. While similar methods have been reported [30,31,32], the use of a lattice path and an intersection-based approach represents a unique contribution of this study. Additionally, the implementation of a coaxial nozzle for the simultaneous extrusion of n-AG and calcium chloride solution introduces a significant advancement in scaffold biofabrication, enabling in situ crosslinking, which was not previously demonstrated in similar approaches.

The ability to create non-planar paths for scaffold deposition has significant implications for biofabrication [33,34], particularly to produce patient-specific scaffolds in regenerative medicine. Traditional 3D printing methods rely on planar deposition, which is inadequate for irregular anatomical geometries [35,36].

The proposed method facilitates the fabrication of scaffolds on irregular surfaces, providing a pathway for personalized medicine and customized tissue engineering solutions. By ensuring precise EE control and consistent material extrusion, this system enables the production of complex tissue-engineered constructs with geometric conformity.

Additionally, the implementation of a coaxial nozzle system enables the simultaneous extrusion of biomaterials and crosslinking agents. The modular design of the EE, which incorporates a LEGO-based support for custom nozzle attachment, offers flexibility and adaptability for other extrusion-based biofabrication processes. This modular approach enhances the potential to incorporate different types of nozzles or multi-material extrusion systems, further broadening the scope of this methodology.

Despite its demonstrated success, this study has several limitations that should be addressed in future work. One notable limitation is the assumption that the anatomical patch remains static during deposition. However, in certain clinical applications, slight shifts or movements of the surface may occur, potentially compromising the accuracy of scaffold deposition. Addressing this issue would require the implementation of real-time feedback control or surface-tracking technology. Another limitation is the use of a fixed interplanar distance of 1 mm to generate the lattice path. While this parameter was sufficient to achieve the desired precision, future research could explore adaptive path generation methods that dynamically adjust the interplanar distance based on surface curvature or anatomical complexity. This approach could increase an accurate path generation.

## 5. Conclusions

This study successfully demonstrates a reproducible MATLAB-based framework for non-planar lattice path generation on irregular anatomical surfaces, overcoming the limitations of traditional planar 3D printing. The kinematic simulation identified an optimal spatial discretization density S ≤ 2.09 mm (N = 30) that achieves positional standard deviation σ = 0.02 mm—below the DOBOT MG400 robotic arm’s 0.05 mm repeatability threshold—balancing trajectory accuracy with computational efficiency. The core accessibly lies in the intersection-based algorithm that slices standard STL files with parallel planes (1 mm spacing) to generate conformable lattice toolpaths using widely available tools, eliminating the need for proprietary software required by existing non-planar approaches. Successful deposition of n-AG biomaterial on humeral head patches confirms practical feasibility and visual geometric conformity to complex anatomies.

## Figures and Tables

**Table 1:** The table presents the mean radius values along the simulated trajectory and the variance as the number of spatial discretization points (N) in the executed path increases.

Number of Points N	Spatial Sampling Step S (mm)	Radius (mm)	Radius (mm)
5	12.56	8.81	1.04
10	6.28	9.68	0.3
20	3.14	9.98	0.15
30	2.09	9.99	0.02

## Data Availability

The data presented in this study are available from the corresponding author upon reasonable request.

## Abbreviations

The following abbreviations are used in this manuscript:

TERM	Tissue engineering and regenerative medicine
Ru	Ruthenium
SPS	Sodium persulfate
GelMA	Gelatin methacryloyl
DW	Deionized water
EE	End-effector
DOF	Degrees of freedom
